# Verses

**Published:** 1907-06

**Authors:** 


### VERSES.

### LOVE'S LABOR.

The dream time, then gleam time,
The night or the day.
Give love to the labor
And life is half play!

—Baltimore Sun.

### HE'S BLUE.

Oh, this world's a good old place,
We are told;
But it isn't, in my case—
I've a cold.

—Denver Post.

### MICROBES.

They say there's microbes in a kiss,
This rumor is not rife;
Come, dear lady, and make of me,
An invalid for life.

—Exchange.

### THE RETORT.

Old Birch, who taught the village school,
Wedded a maid of homespun habit;
He was as stubborn as a mule;
And she as playful as a rabbit,
Poor Kate had scarce become a wife,
Before her husband sought to make her
The pink of country polished life,
And prim and formal as a Quaker.

One day the tutor went abroad,
And simple Katy sadly missed him,

When he returned, behind her lord
She shyly stole, and fondly kissed him,
The husband's anger rose, and red
And white his face alternate grew:
"Less freedom, ma'am!" Kate sighed and said,
"O dear! I did'nt know 'twas you."

—George Pope Morris.

"Dis worl' dat we's a-libin' in is like a cotton row
Where ebery poah nigger hab got his line to hoe,
And ebery time a lazy brudder stops to take a nap,
De gras' keeps on a-growin' for to smudder up his crap.

"When Moses led de Jews across de waters ob de sea,
Dey had to keep a-goin' jis' as fas as fas could be;
And do you 'spouse dat dey could eber hab succeeded in de'er wish
An' reached de promised lan' at las' if dey had stopped to fish?"

—Summary.

THE DEATH OF A FRIEND.

Er good ole frien am gone,
Wev'e laid im een de grabe,
We fresh de flowers from moon ter moon,
An prey de lord e soul ter sabe.

E ben er fren tru life,
Endurn joy er sorrer,
We've had nary scrap ner strife
An e paid back wat e borrer.

Outen dat black skin,
Er soul es wite es snow,
Hab riz, an am now enterin,
Dat Heben, whar all good niggers go.

—B. H. Teague.

